# Quackery

**Published:** 1845-08

**Authors:** 


### QUACKERY.

We clip the following from the Boston Journal of the 16th ult. It points to as fair a specimen of modern impudence as can well be imagined. There is scarcely a town in the United States, however, that does not contain others as veritable as Dodds. We doubt not the sapient "*President*" is ready to confess that a prophet hath no honour in his own country, and very sincerely desires to be saved from his friends, (neighbours!)

*Columbian Mesmeric College.*—A more glaring imposition has not come to light of late, than one that peeps out through the St. Louis (Missouri) papers. One Dodds is there, lecturing, who is styled the *President of the Columbian Mesmeric College and Medical Institution*, established in Boston, *with three professors!* No such institution ever existed here; and if one should appear, the trustees, if wise, would be careful to select a president who could speak his mother tongue grammatically. If the people of St. Louis can swallow such stuff as the president of this imaginary mesmeric college deals in, when not preaching, the druggists will have no more occasion for the sale of ipecac. Really, this is a phenomenon—that animal magnetism, though the silliest system of gullibility extant, should have fallen so low, as to be served up to the Hards and Softs on the Upper Mississippi, by such a man.

